# Optimization of scleroglucan production by *Sclerotium rolfsii* by lowering pH during fermentation via oxalate metabolic pathway manipulation using CRISPR/Cas9

**DOI:** 10.1186/s40694-021-00108-5

**Published:** 2021-02-18

**Authors:** Tianlong Bai, Teng Wang, Yan Li, Na L. Gao, Lixin Zhang, Wei-Hua Chen, Xiushan Yin

**Affiliations:** 1 Applied Biology Laboratory, Shenyang University of Chemical Technology, Shenyang, 110142 China; 2 Key Laboratory of Molecular Biophysics of the Ministry of Education, Hubei Key Laboratory of Bioinformatics and Molecular Imaging, Department of Bioinformatics and Systems Biology, College of Life Science and Technology, Huazhong University of Science and Technology, Wuhan, 430074 China; 3 Liaoning Province Key Laboratory of Green Functional Molecule Design and Development, Shenyang University of Chemical Technology, Shenyang, 110142 China; 4 College of Life Science, HeNan Normal University, Xinxiang, 453007 Henan China

**Keywords:** *AAT1*, CRISPR/Cas9, Oxalate, Scleroglucan, *Sclerotium rolfsii*

## Abstract

**Background:**

*Sclerotium rolfsii* is a potent producer of many secondary metabolites, one of which like scleroglucan is an exopolysaccharide (EPS) appreciated as a multipurpose compound applicable in many industrial fields.

**Results:**

Aspartate transaminase (*AAT1*) catalyzes the interconversion of aspartate and α-ketoglutarate to glutamate and oxaloacetate. We selected *AAT1* in the oxalate metabolic pathway as a target of CRISPR/Cas9. Disruption of *AAT1* leads to the accumulation of oxalate, rather than its conversion to α-ketoglutarate (AKG). Therefore, AAT1-mutant serves to lower the pH (pH 3–4) so as to increase the production of the pH-sensitive metabolite scleroglucan to 21.03 g L^−1^ with a productivity of up to 0.25 g L^−1^·h^−1^.

**Conclusions:**

We established a platform for gene editing that could rapidly generate and select mutants to provide a new beneficial strain of *S. rolfsii* as a scleroglucan hyper-producer, which is expected to reduce the cost of controlling the optimum pH condition in the fermentation industry.

## Background

Microbial biopolymers have gained popularity for use as novel materials substituting plant gums since the past 30 years [1]. The advantages of microbial polysaccharides include the sustainable production and high quality of the product [2]. In addition, EPS obtained from microorganism is easy to recover [3] and hence have become a popular alternative in the recent years. For example, pullulan from *Aureobasidium pullulans*, xanthan from *Xanthomonas sp*., hyaluronic acid from *Streptococcus zooepidemicus*, and scleroglucan from *Sclerotium rolfsii* (Teleomorph: *Athelia rolfsii*) have been reported [4–6]. The molecular weight of scleroglucan is approximately 2–20 × 10^6^ Daltons. Scleroglucan is a polysaccharide composed of a linear chain of β-(1,3)-linked d-glucopyranosyl residues with single β-(1,6)-linked d-glucopyranosyl groups attached to about every third residue of the main chain [7]. Because of its unique structure and a high molecular weight, scleroglucan possesses several beneficial properties for application in oil recovery [8], food industry [9], and the pharmaceutical industry [10]. Scleroglucan is produced mainly via microbial fermentation. Meanwhile, it has several limits, such as the low yield and the high cost of production, which severely hampers scleroglucan application to a wider range of industries [11]. Nevertheless, several excellent properties such as water solubility, pseudoplasticity, moisture retention, salt tolerance, and viscosity stability of scleroglucan deserve resolution of problems encountered in its application.

Other factors such as the phosphate levels or the initial pH does influence scleroglucan production to a much lesser extent [8]. Several researchers have attempted to select the type and concentration of the carbon source to positively influence the production of scleroglucan. Another hindrance is that the mechanism of scleroglucan biosynthesis is not clear. Although some past studies have demonstrated that pH plays a significant role in the fermentation process by affecting the microbial physiology in some ways, such as enzyme activity, cell membrane morphology, nutritional solubility, and ingestion [12], no research has yet reported scleroglucan production increment via manipulation of its relative metabolic pathways at the genomic level.

Oxalic acid is the main acidic metabolite in *S. rolfsi* and in many other fungi, such as *Sclerotinia sclerotiorum* [13]. Oxalic acid has been reported to be directly toxic to plant tissues [14], as it is one of the strong organic acids that is 10,000-times more acidic than acetic acid. Thus, it is believed that oxalic acid can influence the pH in the process of liquid fermentation during scleroglucan production. For generating a new strain of *S. rolfsii* that is more suitable for the fermentation industries, an appropriate adjustment in the oxalate biosynthesis at the genetic level is fast gaining attention toward optimizing the scleroglucan production.

The clustered regularly interspaced short palindromic repeats (CRISPR) and CRISPR-associated (CRISPR-Cas9) system has rapidly progressed as an efficient genome-editing technique in various organisms, including several ascomycetes and several basidiomycete fungi [15]. Currently, there is only limited information available on the manipulation of *S. rolfsii* at the genetic level, owing to the technical limitations such as the lack of well-annotated genome and efficient recombinant DNA methodologies. A few past reports have described transgenic strains based on protoplast transformation [16, 17]. However, there is no report on gene disruption linked with precise phenotype analysis yet. Recently, the draft of *S. rolfsii* genome was published [18]. By combining the transcriptome data [19] and the outcomes of cross-species sequence homology analysis, we could obtain the transcripts for essential enzymes for oxalate synthesis, which offered a roadmap for establishing mutant strains that are of indirect relevance to high-yield production of scleroglucan.

## Results

### CRISPR/Cas9-mediated gene inactivation in* S. rolfsii*

**Fig. 1:**
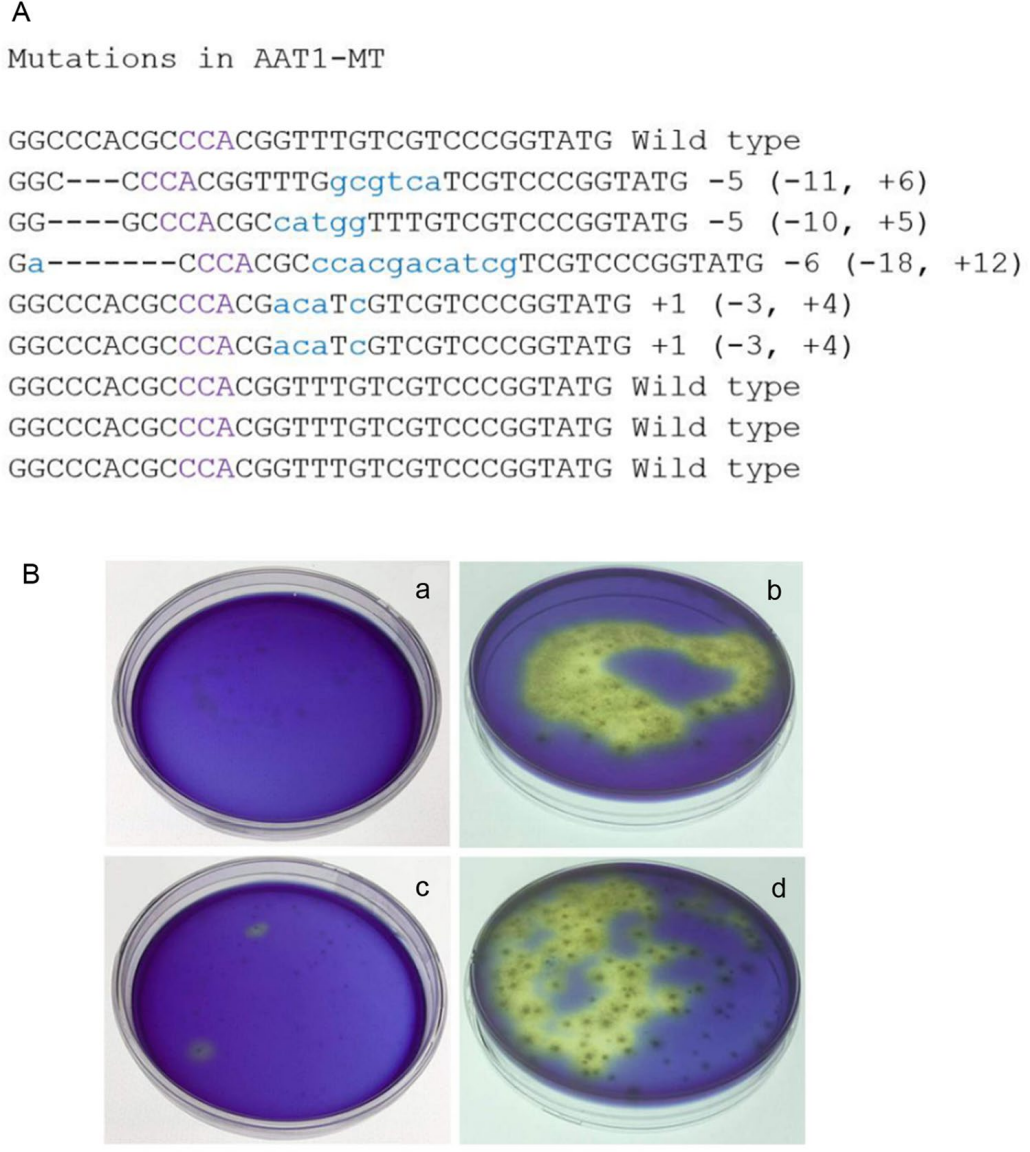
Selection and identification of the mutants by using hygromycin-BPDA medium and sequencing. **A** Sequences of single colonies detected after selection of the hygromycin-BPDA top agar. Deletions are marked by dashes. Insertions and PAMs are marked in blue and purple, respectively. Numbers to the right of the sequences indicate the net loss or gain of bases for each sequence, with the number of bases inserted ( +) or deleted ( −) indicated in parentheses. **B** A rapid method to select mutants compared with the control based on the medium color. Protoplasts of WT cultured in hygromycin-BPDA medium after 5 days (**a**) and 8 days (**b**), respectively. Protoplasts of *S. rolfsii* operated by PEG-mediated transformation of RNPs cultured in hygromycin-BPDA medium after 5 days (**c**) 8 days (**d**), respectively

In order to establish a genetic transformation protocol for *S. rolfsii*, we used the polyethylene glycol (PEG)-based method to transfer plasmid pDHt/sk-PE into the fungus. This plasmid expresses the Cas9 protein labelled with the green fluorescent protein (eGFP) and confers hygromycin resistance. After 5 days of culturing in hygromycin-potato dextrose agar medium, eGFP signal could be clearly detected in *S. rolfsii* hyphae. PCR analysis with eGFP primers also confirmed successful transformation (Additional file 1: Figure S1). To transfer the Cas9 ribonucleoprotein complexes (RNPs) targeting the *AAT1* gene, we employed the PEG-mediated transformation method to deliver RNPs into protoplasts of *S. rolfsii* and used plasmid Htb2-GFP that could only express hygromycin resistance for selection. In order to successfully knock-out *AAT1*, we followed also another approach, whereby we co-transformed Cas9-expressing plasmid pDHt/sk-PE and guide RNA complexes. However, we obtained only one mutant colony. AAT1-mutant (MT) colonies were confirmed via sequencing (Fig. 1a). The AAT1-MT colonies produced more acid metabolites than the wild-type (WT) colonies. The yellow color produced in the hygromycin-BPDA medium as a result of the production of acid metabolites helped in the easy selection of the AAT1-MT colonies from among hundreds of colonies (Fig. 1b). High-performance liquid chromatography–mass spectrometry (HPLC–MS) was used to identify the secreted acid metabolites.

### Disruption of AAT1 leads to the elevation of both oxalic acid and scleroglucan productions

**Fig. 2:**
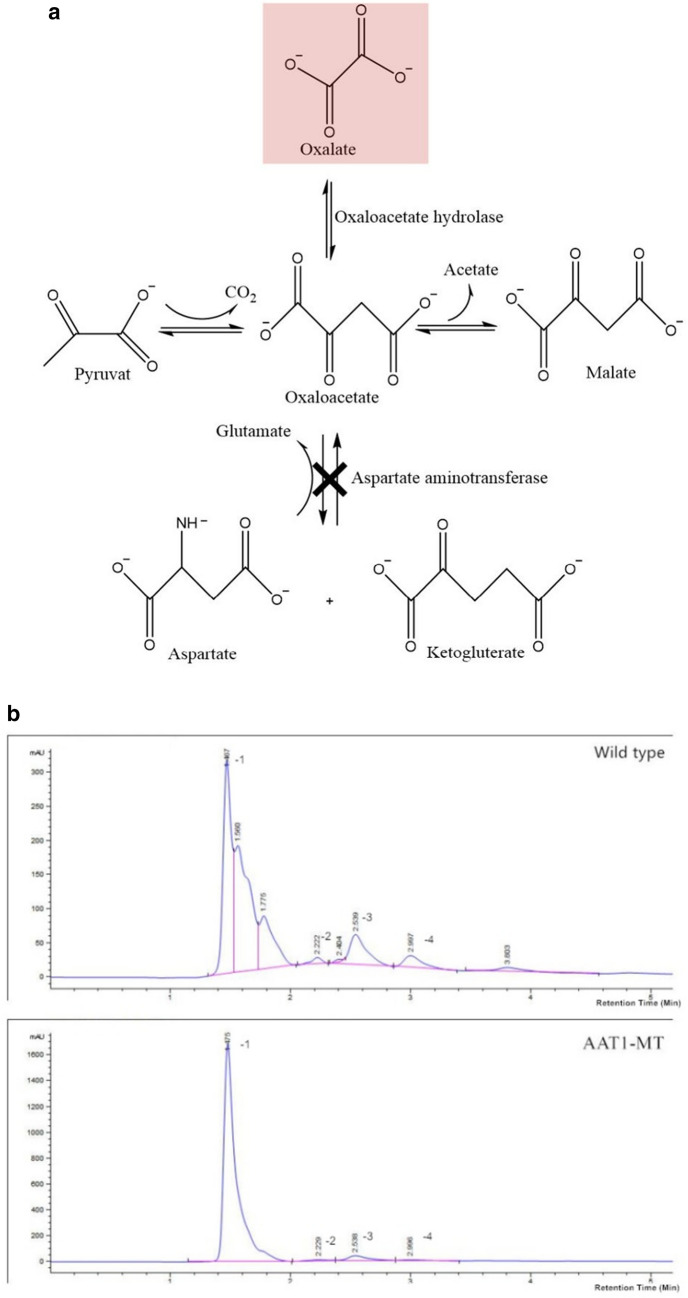

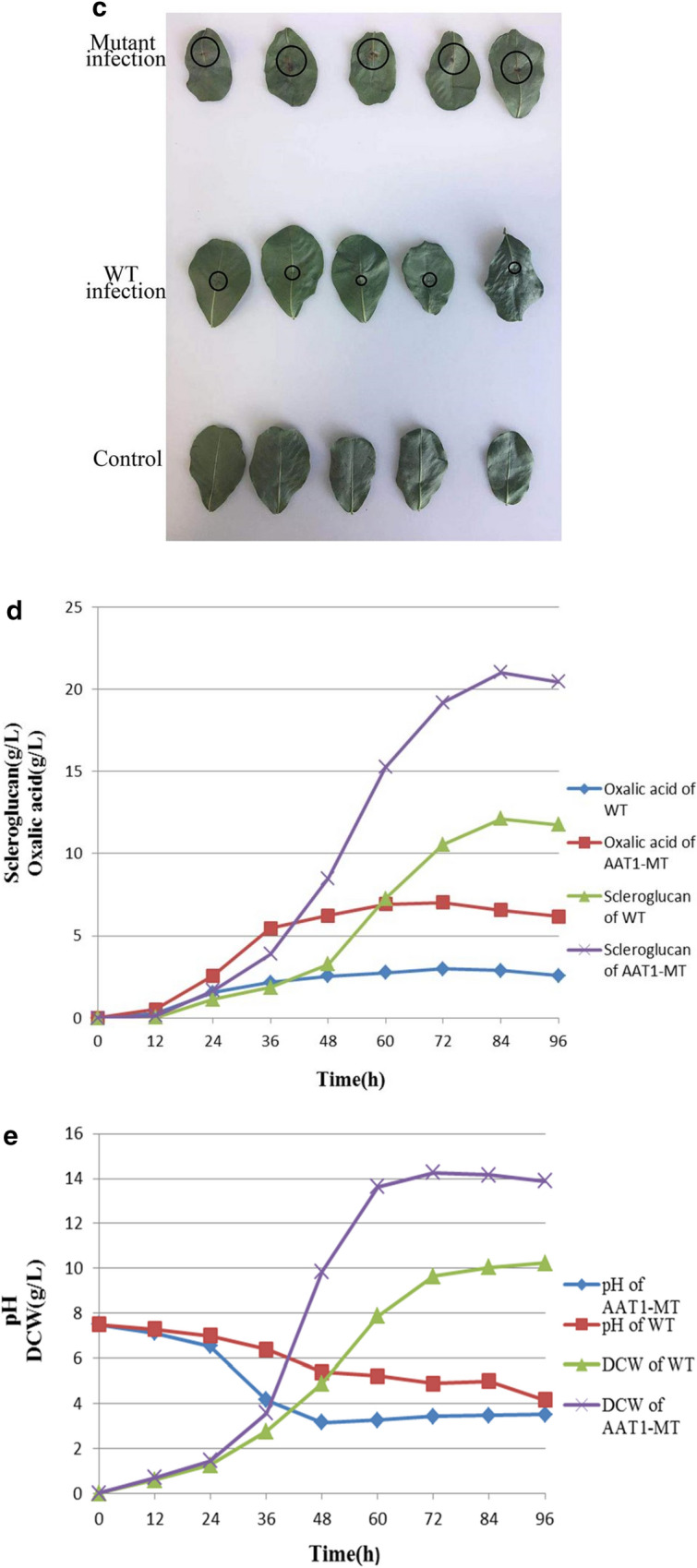
Comparison of the WT and AAT1-MT with respect to oxalic acid production based on bioassays, medium pH, DCW, and scleroglucan production. **a** Metabolic pathway schematic of AAT1-MT. Inactivation of *AAT1* sequence is marked by cross, and the increased oxalic acid production is marked with a red background. **b** Chromatogram of oxalic acid produced by WT and AAT1-MT in liquid medium; peaks were detected at the retention time of 1.4 min. Oxalic acid produced by the mutants was distinctly increased in concentration when compared with that by the WT (peak nos. 1 = oxalic acid, 2 = ferulic acid, 5 = chlorogenic acid, 6 = cinnamic acid). **c** Bioassays of mutants in peanut leaves and controls strains. The lesion area created by the erosion of acid metabolites, which is mainly oxalic acid, showed bright brown color, which is marked by black circles. **d** Scleroglucan and oxalic acid production during bioreactor cultivation. Symbols: AAT1-MT scleroglucan concentration ( ×), WT scleroglucan concentration (▲), AAT1-MT oxalic acid concentration (■), WT oxalic acid concentration (◆). **e** The line chart of pH and DCW concentrations. Symbols: AAT1-MT DCW concentration ( ×), WT DCW concentration (▲), pH of WT (■), pH of AAT1-MT (◆). Each value represents the mean ± standard deviation of measurements from triplicate cultures

The metabolic pathway of AAT1-MT is depicted in Fig. 2a. Based on the measurement of the peak area of oxalic acid (Fig. 2b), we calculated the concentrations of oxalic acid in the WT and AAT1-MT samples to be 843.32 µg mL^−1^ and 2854.42 µg mL^−1^, respectively. In addition, we analyzed α-ketoglutarate (AKG), which is a closely related metabolite in this pathway (Additional file 2: Figure S2). The mass spectrograms are shown in the Additional file 3: Figure S3. Moreover, the mutant strains secreted 3-times more oxalate, but significantly less of AKG, than the WT, indicating that the disruption of *AAT1* deviates the metabolic flow toward oxalate. Clearly, due to the inactivation of Using a plant leave infection assay, we analysed the brownish coloured lesion area on peanut leaves that were provoked by the mutant and wild type strains. As depicted in Fig. 2c, lesions were stronger on the case of the mutant strains when compared to the wild type strains.

The line chart in Fig. 2d on the concentrations of oxalic acid and scleroglucan in the fermentation broth demonstrated that the ability of AAT1-MT for the production of scleroglucan (21.03 g l^−1^) was stronger than that of the WT (12.11 g l^−1^). The scleroglucan production of AAT1-MT increased by 73.66% when compared with that by the WT. The dry cell weight (DCW) and the pH in the fermentation process are shown in Fig. 2e. After 48 h, the pH of the medium could be maintained at approximately 3.37, which slowed down the accumulation of oxalate [20]. This phenomenon may be attributed to the pre-activation of some degradative enzymes, such as oxalate decarboxylase and oxalate oxidase [11], by the high concentration of oxalic acid. In other fungi such as *Coriolus versicofor* and *Collybia velutipes*, the optimum pH of these enzymes has been reported to be 3 [21, 22]. The highest DCW (14.26 g l^−1^) of AAT1-MT was achieved at 72 h, while the DCW of the WT was approximately 10 g^−1^. At a pH of 3–4, both cell growth and polysaccharide synthesis were faster. Furthermore, the scleroglucan productivity of the mutant strain reached 0.25 g/(L h), which was an increase by 78.57% when compared with that obtained from the WT strain (0.14 g/[L h]). After 72 h, the glucose concentration was detected to be approximately 15.6 g/L.

## Discussion

This is the first study to explore the CRISPR-Cas9 system in *S. rolfsii*. In the CRISPR/Cas9 system, the Cas9 endonuclease catalyzes a DNA double-strand break (DSB) aided by a single-guide RNA (sgRNA) containing a 20-nt sequence that matches the sequence upstream of the protospacer-adjacent motif (PAM; 5′-NGG-3′) site on the target locus. The DSB can then be followed by deletion, insertion, or substitution of the sequence using homology recombination or the non-homologous end joining (NHEJ) pathway. Accordingly, we aimed at establish the CRISPR/Cas9 system to alter the metabolic pathway whereby more oxalic acid could be produced to change the pH of the fermentation liquid, which favors scleroglucan production.

Using the Cas9 protein/sgRNA ribonucleoproteins (RNPs) to perform genome editing offers several advantages than with the co-transformation of the Cas9 expression plasmid and sgRNA. One of the major advantages is that the transformation of Cas9 RNPs alleviates the possibility of the integration of a genetic material to a non-targeted region of the genome [23, 24]. In addition, Cas9 and sgRNA can form a stable ribonucleoprotein in vitro, which reduces the likelihood of RNA degradation when compared to that with the Cas9 mRNA/sgRNA transformation [25]. Here, we established the CRISPR-based gene-editing platform in *S. rolfsii*. Combining with a new selective method, that is by using hygromycin-BPDA media to obtain mutants that are different in their ability to change the color of their surroundings, we could efficiently select mutants with altered oxalate production. Moreover, we obtained a mutant that could produce more oxalic acid and scleroglucan as a result of the knockdown of the target gene *AAT1*. However, an increased amount of oxalic acid is not preferred in industrial production. For instance, during industrial scleroglucan production, the formation of the by-product oxalate is undesirable as it lowers the productivity of the process and negatively interferes with the downstream processing of scleroglucan. In addition, the mutant strain grows faster than the WT in the presence of excess oxalate, which could make the environment more suitable for survival and secretion. It is therefore necessary to add an appropriate stabilizer, such as phosphate or citrate, that can react with oxalic acid to stabilize the pH of the system.

## Conclusions

Our results provide an effective strategy to indirectly control the pH condition for increasing the yield of sclerogulcan in industrial production by using a newly emerging gene-editing tool CRISPR/ Cas9. Our study demonstrates the potential strategy to radically decrease the cost of artificially regulating pH during the industrial sclerogulcan fermentation process.

## Methods

### Strain and culture condition

*S. rolfsii* (Teleomorph: *Athelia rolfsii*) deposited in the Chinese Academy of Agricultural Sciences (CAAS) was used as the WT and cultured in PDA medium (composed of peeled potato 200 g, dextrose 20 g, agar 15 g, distilled water 1 L, pH = 7.5) and in a fermentation medium (composed of glucose 130 g, NaNO_3_ 3 g, yeast extract 1 g, KCl 0.5 g, KH_2_PO_4_ 1 g, MgSO_4_·7H_2_O 0.5 g, distilled water 1 L, pH = 7.5) at 30 °C. Batch fermentations were performed in a 5-L fermenter containing 3-L of the fermentation medium. All the components were autoclaved for 20 min at 115 °C for sterilization. Hygromycin-BPDA medium was mainly composed of PDA and hygromycin (35 µg ml^−1^) with bromophenol blue (60 µg ml^−1^), which is an indicator that changes color from yellow to blue at the pH of 3–4.6. The strain was identified with ITS primers. All primers used in this study are listed in the Additional file 4: Table S1.

### AAT1 identification

To identify *AAT1*, we first downloaded the assembled genome of *Athelia rolfsii* (GCA_000961905.2) from NCBI and annotated its protein-coding genes using the GeneMark-ES [26] with opinions ‘–ES –fungus –sequence’. We also assembled a transcriptome data available from the NCBI SRA database for *A. rolfsii* (accession ID: SRS025455) using SPAdes [27] with opinions ‘–sc -s –careful -k 75′, as well as annotated the protein-coding genes. All protein-coding genes were combined and renamed, starting with A0000000.

We manually selected *AAT1* from 6 well-annotated fungal genomes including *Scheffersomyces stipitis, Emiliania huxleyi, Kluyveromyces marxianus, Saccharomyces cerevisiae, Pseudogymnoascus destructans*, *and Candida albicans* and BLASTed their protein sequences against the identified protein-coding genes of *A. rolfsii* (Additional file 5: Figure S4a). We identified the gene “A0001768” from the transcriptome data as the best hit. We then confirmed that “A0001768” could cover the full-length of *AAT1* by building a multiple sequence alignment using the protein sequences of “A0001768” and the 6 fungal *AAT1* proteins using an online version of the Clustal Omega [28, 29]. The result could be visualized using an online version of Mview [30] later.

Finally, we obtained the gene structure (i.e., the delineation of its exons) of “A0001768” (Additional file 5: Figure S4b) by mapping its coding sequences to its assembled genome using the BLAT [31].

### Preparation of Cas9 protein, sgRNA, and plasmids

Cas9 protein was purchased from New England BioLabs Inc. The protospacer sequences (CAGACCGGGACGACAAACCGTGG) of sgRNA named “CR1-AAT1” were designed and screened against the target by using the Geneious software and confirmed with the online tool CHOPCHOP [32, 33]. TrancrRNA and crRNA were purchased from GenePharma (Suzhou China). The assembling of guide-RNA complexes was performed as described here: 4.5 μL of crRNA (50 μM), 4.5 μL of trancrRNA (50 μM), and 10 μL of duplex buffer were mixed in a well, and the well was incubated at 95 °C for 5 min and then cooled down to the room temperature for 20 min. All plasmids used in this study and their purposes are listed in the Additional file 6: Table S2, and they were all purchased from Addgene.

### Preparation of protoplast

The protoplast formation was performed as described elsewhere [34], with some slight modifications. Briefly, after 90 min of the incubation for depriving the cell wall, we used sterile 100 μm of the Cell Strainer to filter out the impurities of the reaction mixture.

### Transformation for S. rolfsii

PEG-mediated fungal transformation was conducted according to the previously described method [35], with some modifications. Briefly, the RNPs and Htb2-GFP plasmid were prepared during the generation of the protoplasts, where the Cas9 RNPs were prepared as follows: 10 μL of assembled guide-RNA complexes (as described above) and 5 μL of Cas9 protein (50 μM) were added to a total volume of 50 μL with 5 μL of 10 × Cas9 Nuclease Reaction Buffer and diethylpyrocarbonate (DEPC)-treated water. This mixture was incubated in a 37 °C water bath for 25 min, and 100 µL of the fungal protoplasts were mixed with 20 μL of Cas9 RNPs and Htb2-GFP plasmid at the room temperature for 20 min. Then, 40% of PEG was added to the above system, followed by incubation at the room temperature for 20 min. After STC buffer (composed of 1.2 M Sorbitol, 10 mM Tris–HCl, 10 mM CaCl_2_; pH 7.5) was mixed well by gently inverting the tubes several times, the total system was directly transferred into the MGY regeneration medium (composed of 1% malt extract, 1% glucose, 0.1% yeast extract, 2% agar; pH 5.5) with 0.5 M sucrose osmotic stabilizer. After 4 days of incubation, the protoplasts developed into incipient colonies that could be observed with the naked eye, and the bottom agar was covered with 20-mL of top-selective hygromycin-BPDA agar.

### Fluorescence microscopy

The subcellular localization of eGFP was followed using the Leica DMi8 Fluorescence microscope. The transformants containing pDHt/sk-PE were cultured in the MGY agar plate in a dark incubator at 30 °C for 7 days.

### Analytical method

Mycelium was obtained by germination of water-preserved sclerotia on PDA agar plate and incubated at 30 °C, as previously described [36]. Then, two 250-mL of Erlenmeyer flasks containing 50-mL of the liquid MGY medium were inoculated with 5 mycelium-covered agar discs (approximately 5-mm diameter) removed from the 2-day-old PDA culture of WT and AAT1-MT, respectively, at 30 °C on an orbital shaker at 250 rev min^−1^ for 45 h, followed by HPLC–MS analysis. The mycelia were frozen in the refrigerator at − 80 °C. After thawing, the mycelia were grinded in a mortar until they were completely broken and mushy in texture. Then, equal volume of ethyl acetate was added to extract and collect the ethyl acetate phase under the ultrasonic condition at 100 kHz for 1 h. Rotatory evaporation was performed to dry the collected phase at 55 ℃, after which 6–mL of methanol was added into a volumetric flask, followed by testing of the metabolites from the mycelium, such as AKG. The HPLC system (Agilent Technologies Inc., California, United States of America) was coupled with an MS detector (AB SCIEX, Foster City, CA, USA) equipped with electrospray ionization (ESI) source with positive and negative modes (ESI + and ESI −). Reverse-phase chromatographic analysis was performed using a C-18 reverse-phase HPLC column (200 mm × 4.6 mm internal diameter, 5-μm particle size) at 25 °C under isocratic conditions, where the concentration of the mobile phase was kept constant throughout the run. The running conditions included a 10-μL injection volume of the mobile phase methanol-0.02% acid ([NH_4_]2HPO4) (5:95, v/v), at the flow rate of 0.8 mL min^−1^, and detected at 197 nm. The samples were filtered through a membrane filter (0.22-μm pore size; ANPEL) prior to injection in a sample loop. The standard curve and the equation of linear regression are depicted in Additional file 7: Figure S5. The peak areas of all oxalic acid relative standards and samples are listed in Additional file 8: Table S3.

We also comparatively measured the scleroglucan production in the fermentation broth between the WT and AAT1-MT. The fermentation broth was diluted 5-times with distilled water, heated at 70 °C for 40 min, and then centrifuged at 13,400×*g* for 25 min. The precipitate obtained was washed with distilled water and dried at 105 °C. An equal volume of absolute ethanol was added to the supernatant in order to precipitate scleroglucan. The mixture was then maintained on an ice bath for 12 h to precipitate completely. Finally, the scleroglucan produced was recovered by vacuum drying [37].

### Bioassays of acid metabolites

In order to identify whether AAT1-MT could produce more acid metabolites than WT, bioassays were performed using detached peanut leaflets inoculated with an agar plug of *S. rolfsii* mycelia. The *S. rolfsii* cultures were grown on PDA plates and 5-mm plugs were taken from the actively growing edge. Leaflets were wounded with a knife over an area of 5 mm on the adaxial surface, near the midvein, and the removed plugs were placed on the open wounds. Five leaflets were inoculated for each plant line tested using a minimal quantity of agar in each plug. The plates were then incubated for 36 h at 30 °C, after which the lesion area could be detected by the development of bright brown color caused by reaction with oxalic acid.

### Statistical analysis

All experiments with the WT and AAT1-MT mycelium samples were performed in three biological replicates. The data obtained from repeated HPLC analyses were pooled and subjected to analysis of variance (ANOVA) for statistical significance by the least significance difference (LSD) test at P = 0.01. An independent sample *t*-test was performed for statistical evaluations between the WT and AAT1-MT strains (P ≤ 0.05) by using the SPSS 21.0 software (IBM, Chicago, IL, USA).

## Supplementary Information

**Additional file 1: Figure. S1.** Identification of the transformation for pDHt/sk-PE plasmid.

**Additional file 2: Figure. S2.** Comparison of WT and AAT1-MT with respect to AKG level.

**Additional file 3: Figure. S3.** Determination by mass spectrograms of peaks related to oxalic acid and AKG.

**Additional file 4: Table S1.** Primers used in this study.

**Additional file 5: Figure. S4.** Multiple sequence alignment and gene structure of the *AAT1* gene.

**Additional file 6: Table S2.** Plasmids and their purposes.

**Additional file 7: Figure. S5.** Standard curve and equation of linear regression.

**Additional file 8: Table S3.** The peak areas of all oxalic acid relative standards and samples.

## Data Availability

Not applicable.

## Abbreviations

AKG: α-Ketoglutarate
CRISPR: Clustered regularly interspaced short palindromic repeats
crRNA: CRISPR-RNA
DCW: Dry cell weight
DSB: Double-strand break
EPS: Exopolysaccharide
gRNA: Guide RNA
NHEJ: Non-homologous end joining
PAM: Protospacer adjacent motif
sgRNA: Single guide RNA
RNPs: Ribonucleoprotein complexes

## References

[1] Paul F, Morin A, Monsan P (1986). Microbial polysaccharides with actual potential industrial applications. Biotechnol Adv.

[2] Freitas F, Torres CAV, Reis MAM (2017). Engineering aspects of microbial exopolysaccharide production. Bioresour Technol.

[3] Donot F, Fontana A, Baccou JC, Schorr-Galindo S (2012). Microbial exopolysaccharides: main examples of synthesis, excretion, genetics and extraction. Carbohydr Polym.

[4] Badle SS, Jayaraman G, Ramachandran KB (2014). Ratio of intracellular precursors concentration and their flux influences hyaluronic acid molecular weight in *Streptococcus zooepidemicus* and recombinant *Lactococcus lactis*. Bioresour Technol.

[5] Brumano LP, Antunes FAF, Souto SG, Dos Santos JC, Venus J, Schneider R, da Silva SS (2017). Biosurfactant production by *Aureobasidium pullulans* in stirred tank bioreactor: new approach to understand the influence of important variables in the process. Bioresour Technol.

[6] Wang Z, Wu J, Zhu L, Zhan X (2016). Activation of glycerol metabolism in *Xanthomonas campestris* by adaptive evolution to produce a high-transparency and low-viscosity xanthan gum from glycerol. Bioresour Technol.

[7] Farina JI, Sineriz F, Molina OE, Perotti NI (1998). High scleroglucan production by *Sclerotium rolfsii*: in fluence of medium composition. Biotechnol Lett.

[8] Schmid J, Meyer V, Sieber V (2011). Scleroglucan: biosynthesis, production and application of a versatile hydrocolloid. Appl Microbiol Biotechnol.

[9] Castillo NA, Valdez AL, Farina JI (2015). Microbial production of scleroglucan and downstream processing. Front Microbiol.

[10] Coviello T, Palleschi A, Grassi M, Matricardi P, Bocchinfuso G, Alhaique F (2005). Scleroglucan: aversatile polysaccharide for modified drug delivery. Molecules.

[11] Tan R, Lyu Y, Zeng W, Zhou J (2019). Enhancing scleroglucan production by *Sclerotium rolfsii* WSH-G01 through a pH-shift strategy based on kinetic analysis. Bioresour Technol.

[12] Schilling BM, Henning A, Rau U (2000). Repression of oxalic acid biosynthesis in the unsterile scleroglucan production process with *Sclerotium rolfsii* ATCC 15205. Bioprocess Eng.

[13] Monazzah M, Rabiei Z, Enferadi ST (2018). The effect of oxalic acid, the pathogenicity factor of *Sclerotinia Sclerotiorum* on the two susceptible and moderately resistant lines of sunflower. Iran J Biotechnol.

[14] Kyoung SK, Min JY, Dickman MB (2008). Oxalic acid is an elicitor of plant programmed cell death during *Sclerotinia Sclerotiorum* disease development. Mol Plant Microbe Interact.

[15] Kunitake E, Tanaka T, Ueda H, Endo A, Yarimizu T, Katoh E, Kitamoto H (2019). CRISPR/Cas9-mediated gene replacement in the basidiomycetous yeast *Pseudozyma antarctica*. Fungal Genet Biol.

[16] Yasokawa D, Shimizu T, Nakagawa R, Ikeda T, Nagashima K (2003). Cloning, sequencing, and heterologous expression of a cellobiohydrolase cDNA from the basidiomycete *Corticium rolfsii*. Biosci Biotechnol Biochem.

[17] Sharma M, Schmid M, Rothballer M, Hause G, Zuccaro A, Imani J, Kämpfer P, Domann E, Schäfer P, Hartmann A, Kogel KH (2008). Detection and identification of bacteria intimately associated with fungi of the order Sebacinales. Cell Microbiol.

[18] Iquebal MA, Tomar RS, Parakhia MV, Singla D, Jaiswal S, Rathod VM, Padhiyar SM, Kumar N, Rai A, Kumar D (2017). Draft whole genome sequence of groundnut stem rot fungus *Athelia rolfsii* revealing genetic architect of its pathogenicity and virulence. Sci Rep..

[19] Schmid J, Muller-Hagen D, Bekel T, Funk L, Stahl U, Sieber V, Meyer V (2010). Transcriptome sequencing and comparative transcriptome analysis of the scleroglucan producer *Sclerotium rolfsii*. BMC Genomics.

[20] Culbertson BJ, Krone J, Gatebe E, Furumo NC, Daniel SL (2007). Impact of carbon sources on growth and oxalate synthesis by the phytopathogenic fungus *Sclerotinia sclerotiorum*. World J Microbiol Biotechnol.

[21] Dutton MV, Evans CS, Atkey PT, Wood DA (1993). Oxalate production by Basidiomycetes, including the white-rot species Coriolus versicolor and *Phanerochaete chrysosporium*. Appl Microbiol Biotechnol.

[22] Kuan IC, Tien M (1993). Stimulation of Mn-peroxidase activity – a possible role for oxalate in lignin biodegradation. PNAS.

[23] Longmuir S, Akhtar N, MacNeill SA (2019). Unexpected insertion of carrier DNA sequences into the fission yeast genome during CRISPR-Cas9 mediated gene deletion. BMC Res Notes.

[24] Skryabin BV, Kummerfeld DM, Gubar L (2020). Pervasive head-to-tail insertions of DNA templates mask desired CRISPR-Cas9-mediated genome editing events. Sci Adv..

[25] Shi TQ, Liu GN, Ji RY, Shi K, Song P, Ren LJ, Huang H, Ji XJ (2017). CRISPR/Cas9-based genome editing of the filamentous fungi: the state of the art. Appl Microbiol Biotechnol.

[26] Ter-Hovhannisyan V, Lomsadze A, Chernoff YO, Borodovsky M (2008). Gene prediction in novel fungal genomes using an ab initio algorithm with unsupervised training. Genome Res.

[27] Bankevich A, Nurk S, Antipov D (2012). SPAdes: a new genome assembly algorithm and its applications to single-cell sequencing. J Comput Biol.

[28] Sievers F, Wilm A, Dineen D, Gibson TJ, Karplus K, Li W, Lopez R, McWilliam H, Remmert M, Söding J, Thompson JD, Higgins DG (2011). Fast, scalable generation of high-quality protein multiple sequence alignments using Clustal Omega. Mol Syst Biol.

[29] Madeira F, Park YM, Lee J, Buso N, Gur T, Madhusoodanan N, Basutkar P, Tivey ARN, Potter SC, Finn RD, Lopez R (2019). The EMBL-EBI search and sequence analysis tools APIs in 2019. Nucleic Acids Res.

[30] Brown NP, Leroy C, Sander C (1998). MView: a web-compatible database search ormultiple alignment viewer. Bioinformatics.

[31] Kent WJ (2002). BLAT–the BLAST-like alignment tool. Genome Res.

[32] Labun K, Montague TG, Gagnon JA, Thyme SB, Valen E (2016). CHOPCHOP v2: a web tool for the next generation of CRISPR genome engineering. Nucleic Acids Res.

[33] Lee CM, Cradick TJ, Fine EJ, Bao G (2016). Nuclease target site selection for maximizing on-target activity and minimizing off-target effects in genome editing. Mol Ther NLM.

[34] Farina JI, Molina OE, Figueroa LI (2004). Formation and regeneration of protoplasts in *Sclerotium rolfsii* ATCC 201126. J Appl Microbiol.

[35] Liu Z, Friesen TL (2012). Polyethylene glycol (PEG)-mediated transformation in filamentous fungal pathogens. Methods Mol Biol.

[36] Fariña JI, Siñeriz F, Molina OE, Perotti NI (1996). Low-cost method for the preservation of *Sclerotium rolfsii* Proimi F-6656: Inoculum standardization and its use in scleroglucan production. Biotechnol Tech.

[37] Survase SA, Saudagar PS, Singhal RS (2007). Use of complex media for the production of scleroglucan by *Sclerotium rolfsii* MTCC 2156. Bioresour Technol.

