# Supplementary material for: Optimization of scleroglucan production by Sclerotium rolfsii by lowering pH during fermentation via oxalate metabolic pathway manipulation using CRISPR/Cas9
Source: Fungal Biol Biotechnol. 2021 Feb 18;8:1. doi: 10.1186/s40694-021-00108-5 (PMC7893912; doi:10.1186/s40694-021-00108-5)

**Supplementary fig. S1** Identification of the transformation for pDHt/sk-PE plasmid

(A) GFP-1 and GFP-2 were selected as the transformants by hygromycin-PDA top agar that showed 400-bp band with the eGFP primers; however, the WT-1 and WT-2 did not. (B) (a) A WT screened by microscope. (b-d) Three single colonies of the transformants. Hyphae of WT could barely show green filamentous fluorescent, except for the background signal from the agar. In contrast, bright GFP fluorescent signal was detected for the transformants.


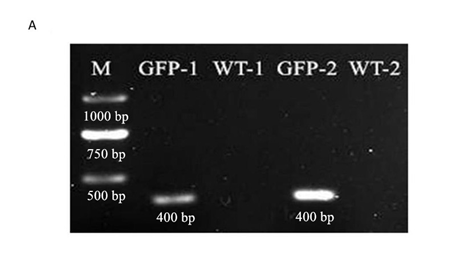


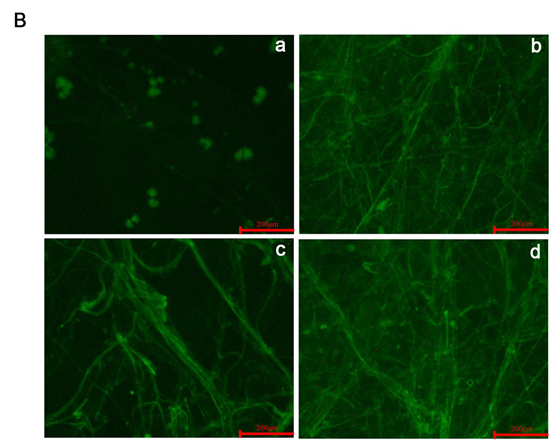

Supplement: Supplementary file 1 — Additional file 1: Figure. S1. Identification of the transformation for pDHt/sk-PE plasmid. [file 40694_2021_108_MOESM1_ESM.docx]
