# Supplementary material for: Optimization of scleroglucan production by Sclerotium rolfsii by lowering pH during fermentation via oxalate metabolic pathway manipulation using CRISPR/Cas9
Source: Fungal Biol Biotechnol. 2021 Feb 18;8:1. doi: 10.1186/s40694-021-00108-5 (PMC7893912; doi:10.1186/s40694-021-00108-5)
Supplement: Supplementary file 2 — Additional file 2: Figure. S2. Comparison of WT and AAT1-MT with respect to AKG level. [file 40694_2021_108_MOESM2_ESM.docx]

**Supplementary fig. S2** Comparison of WT and AAT1-MT with respect to AKG level

(a) HPLC analysis of standard solutions of AKG showed peaks atretention time 1.5 min. (b and c) Compared with the extraction of WT (Figure S3 b), the extraction AAT1-MT (Figure S3 c) showed decreased level of AKG.


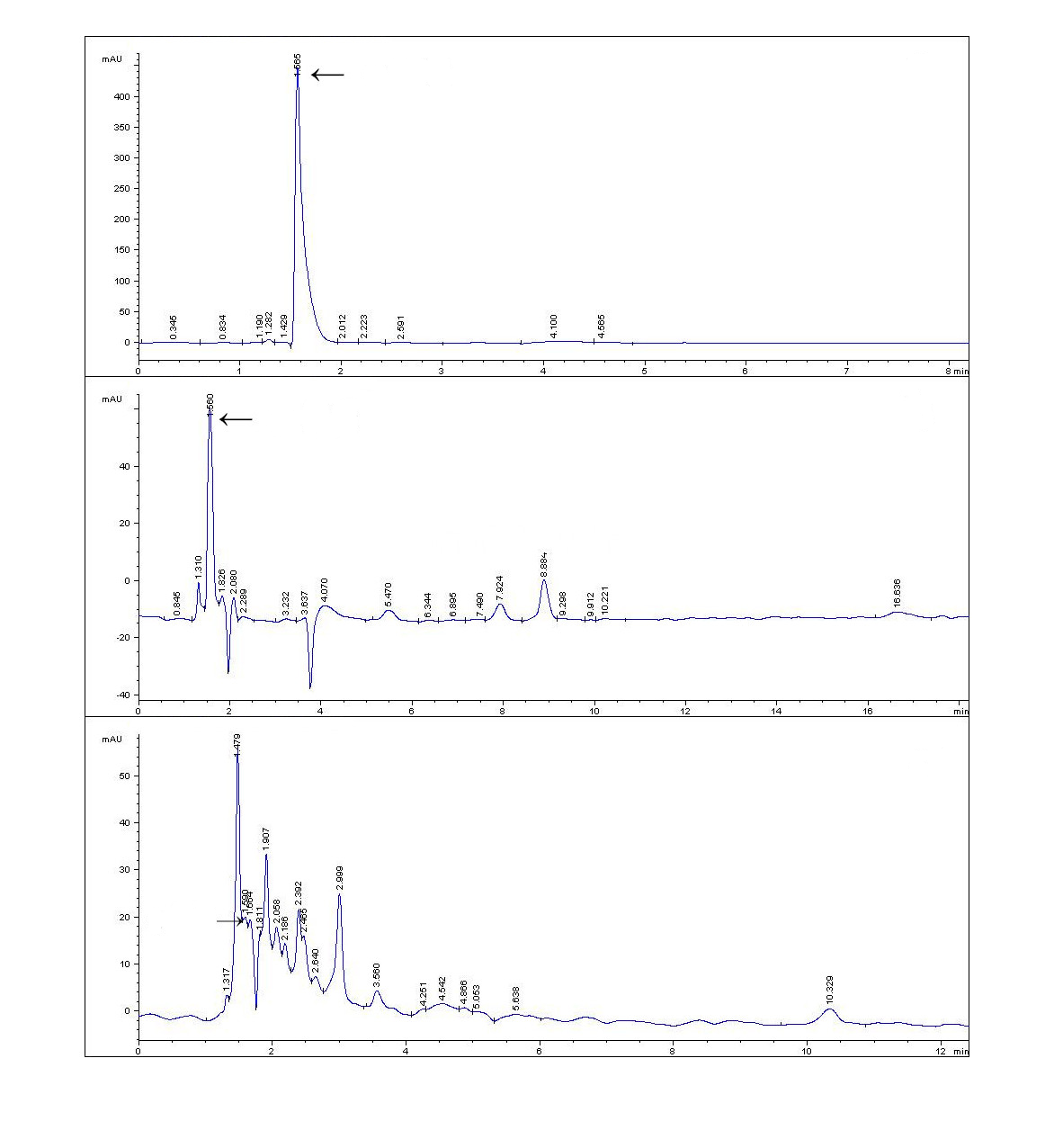


a

AKG

Standards

b

AKG

WT

c

AKG

AAT1-MT
