# Supplementary material for: Optimization of scleroglucan production by Sclerotium rolfsii by lowering pH during fermentation via oxalate metabolic pathway manipulation using CRISPR/Cas9
Source: Fungal Biol Biotechnol. 2021 Feb 18;8:1. doi: 10.1186/s40694-021-00108-5 (PMC7893912; doi:10.1186/s40694-021-00108-5)
Supplement: Supplementary file 3 — Additional file 3: Figure. S3. Determination by mass spectrograms of peaks related to oxalic acid and AKG. [file 40694_2021_108_MOESM3_ESM.docx]

**Supplementary fig. S3** Determination by Mass spectrograms of peaks related to oxalic acid and AKG

(a and b) Mass spectrograms of WT and AAT1-MT o**xalic acid relative peaks are acquired in negative ionization mode (**ESI−)**. (c and d)** Mass spectrograms of WT and AAT1-MT AKG **relative peaks are acquired in positive ionization mode (**ESI+)**. HPLC-MS analysis further showed that the oxalic acid and AKG with [M-H]^-^ m/z 89 and [M+Na]^+^ m/z 169, respectively.**


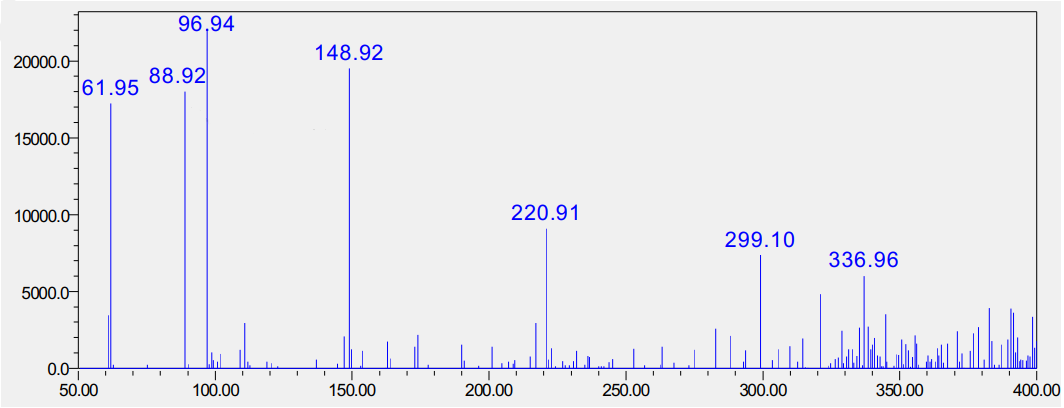


a

WT

Oxalic acid

→


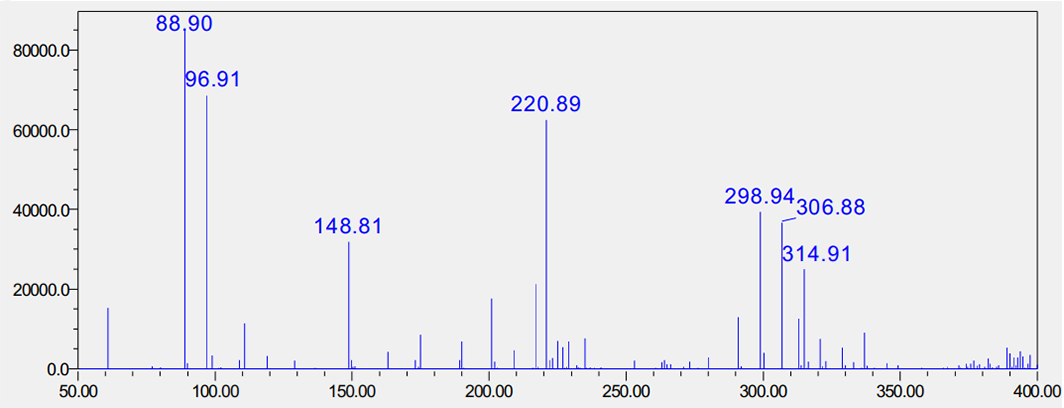


b

Oxalic acid

→

AAT1-MT


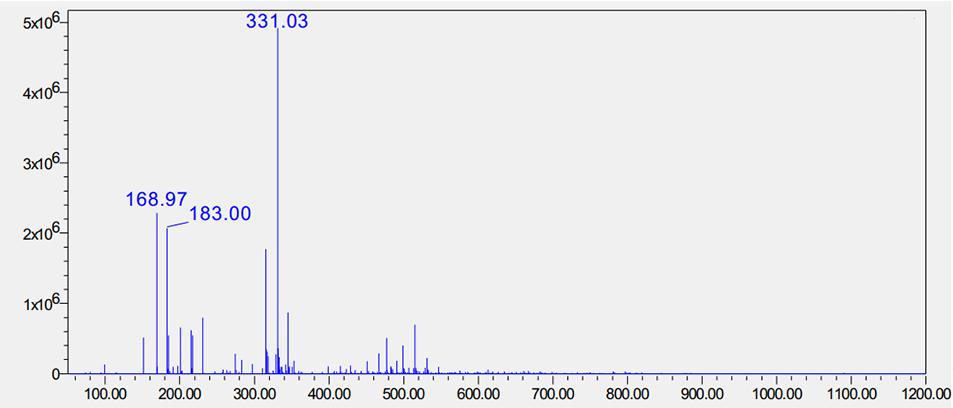


c

WT

AKG

→


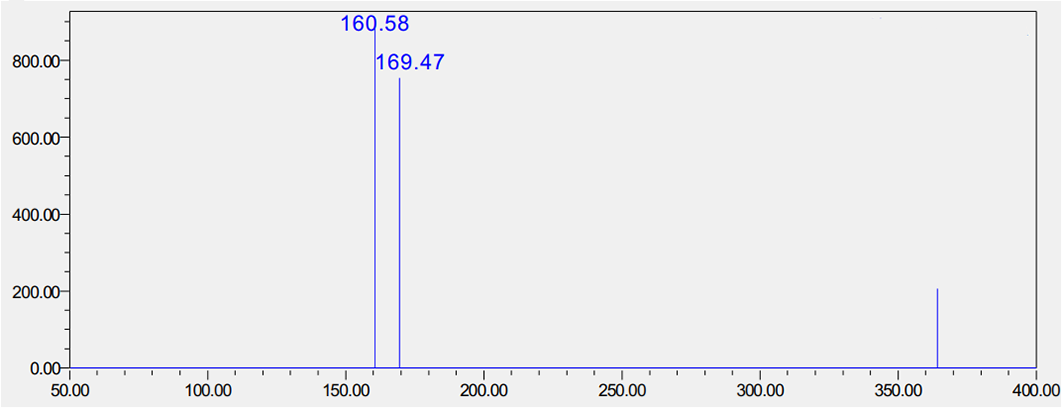


d

AKG

→

AAT1-MT
