# Supplementary material for: Optimization of scleroglucan production by Sclerotium rolfsii by lowering pH during fermentation via oxalate metabolic pathway manipulation using CRISPR/Cas9
Source: Fungal Biol Biotechnol. 2021 Feb 18;8:1. doi: 10.1186/s40694-021-00108-5 (PMC7893912; doi:10.1186/s40694-021-00108-5)
Supplement: Supplementary file 4 — Additional file 4: Table S1. Primers used in this study. [file 40694_2021_108_MOESM4_ESM.docx]

**Table S1** Primers used in this study

| Primer name | Sequence 5′–3′ |
| --- | --- |
| ITS1 | 5’-CCGTAGGTGAACCTGCGG-3’ |
| ITS4 | 5’-TCCTCCGCTTATTGATATGC-3’ |
| OX-f | 5’-TCGATATCCCTTCCTCCAC-3’ |
| OX-r | 5’-AGTGGTGGCAGCACCACTAAAG-3’ |
| AAT1-f | 5’-TTCAAGCAGAAAACTGGAATG-3' |
| AAT1-r | 5’-ATTGGTAAAACTTGCTGAG-3’ |
| GFP-f | 5'-CGACGTAAACGGCCACAAG-3' |
| GFP-r | 5'-CTGTCGGCCATGATATA-3' |
