# Supplementary material for: Optimization of scleroglucan production by Sclerotium rolfsii by lowering pH during fermentation via oxalate metabolic pathway manipulation using CRISPR/Cas9
Source: Fungal Biol Biotechnol. 2021 Feb 18;8:1. doi: 10.1186/s40694-021-00108-5 (PMC7893912; doi:10.1186/s40694-021-00108-5)

**Supplementary fig. S4 a** Multiple sequence alignment and gene structure of the AAT1 gene.

Fig. s1 a Multiple sequence alignment with the six fungal AAT1 proteins

The multiple sequence alignment of the protein sequences of “A0001768” and the six fungal AAT1 proteins built by an online version of Clustal Omega and visualized by Mview using *K. marxianus* as a reference sequence. Each row shows the coverage, percent identity and residues of alignment that seven protein sequences with the reference genome. Only those residues that are identical to the reference sequence can be colored.


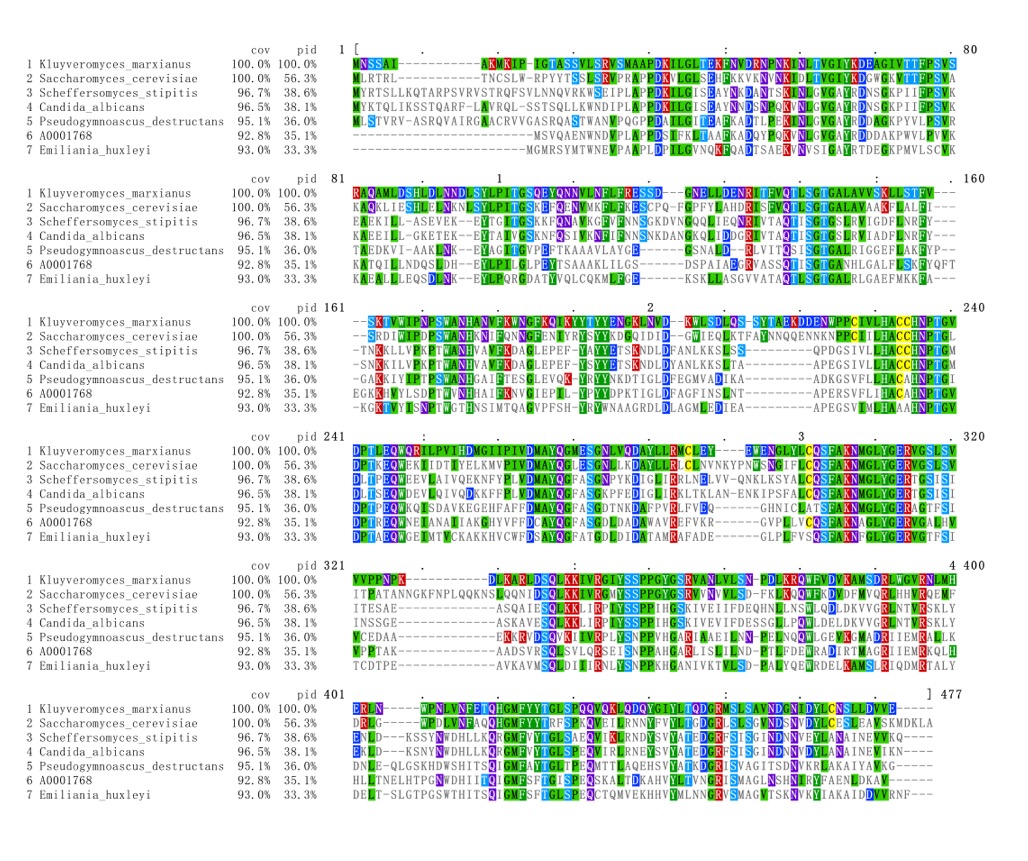


**Supplementary fig. S1 b** **Gene structure of the AAT1 gene**

Delineation of the AAT1 gene (A0001768) into seven exons was obtained by aligning the coding region of A0001768 against the assembled genome of *A. rolfsii* obtained from NCBI (accession ID: GCA_000961905.2). The black boxes indicate exons, and the red line under exon2 indicates the gRNA. The numbers above the exons indicate their positions on the coding region.


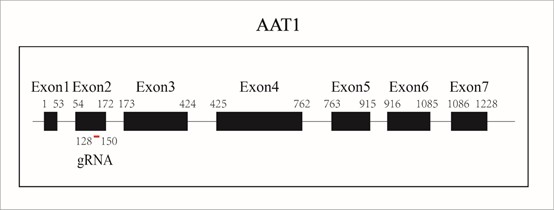

Supplement: Supplementary file 5 — Additional file 5: Figure. S4. Multiple sequence alignment and gene structure of the AAT1 gene. [file 40694_2021_108_MOESM5_ESM.docx]
