# Supplementary material for: Optimization of scleroglucan production by Sclerotium rolfsii by lowering pH during fermentation via oxalate metabolic pathway manipulation using CRISPR/Cas9
Source: Fungal Biol Biotechnol. 2021 Feb 18;8:1. doi: 10.1186/s40694-021-00108-5 (PMC7893912; doi:10.1186/s40694-021-00108-5)
Supplement: Supplementary file 6 — Additional file 6: Table S2. Plasmids and their purposes. [file 40694_2021_108_MOESM6_ESM.docx]

**Table S2** Plasmids and their purposes

| Plasmid name | Purpose |
| --- | --- |
| pDHt/sk-PE (addgene #92126) | Express Cas9 protein , eGFP and hygromycin resistance |
| Htb2-GFP (addgene #117669) | Express hygromycin resistance |
