# Supplementary material for: Optimization of scleroglucan production by Sclerotium rolfsii by lowering pH during fermentation via oxalate metabolic pathway manipulation using CRISPR/Cas9
Source: Fungal Biol Biotechnol. 2021 Feb 18;8:1. doi: 10.1186/s40694-021-00108-5 (PMC7893912; doi:10.1186/s40694-021-00108-5)
Supplement: Supplementary file 7 — Additional file 7: Figure. S5. Standard curve and equation of linear regression. [file 40694_2021_108_MOESM7_ESM.docx]

**Supplementary fig. S5** Standard curve and equation of linear regression

Equation of linear regression was generated by drawing the standard curve with the concentration of oxalic acid standards（µg/mL） as the abscissa and the chromatographic peak area (µAU.s) as the ordinate. Standard oxalic acid solutions were prepared more appropriately with concentrations of 800 µg/mL, 900 µg/mL, 1000 µg/mL, 2000 µg/mL and 3000 µg/mL for further precise calculation of concentrations of WT and AAT1-MT with external standard method.

**
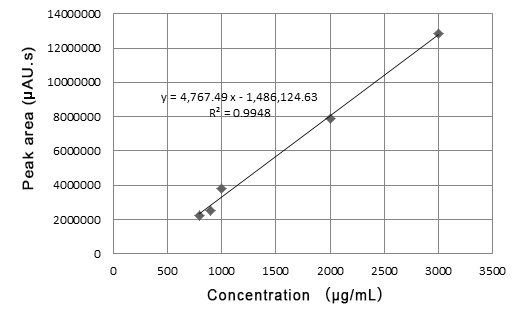
**
