# Supplementary material for: Optimization of scleroglucan production by Sclerotium rolfsii by lowering pH during fermentation via oxalate metabolic pathway manipulation using CRISPR/Cas9
Source: Fungal Biol Biotechnol. 2021 Feb 18;8:1. doi: 10.1186/s40694-021-00108-5 (PMC7893912; doi:10.1186/s40694-021-00108-5)
Supplement: Supplementary file 8 — Additional file 8: Table S3. The peak areas of all oxalic acid relative standards and samples. [file 40694_2021_108_MOESM8_ESM.docx]

**Table S3** The peak areas of all oxalic acid relative standards and samples

| Concentration (µg ml^-1^) | Peak area (µAU.s) |
| --- | --- |
| 800 | 2207131 |
| 867 (WT) | 2326636 |
| 900 | 2488549 |
| 1000 | 3817236 |
| 2000 | 7906438 |
| 2807 (AAT1 -MT) | 12235626 |
| 3000 | 12859658 |
